# Supplementary material for: Identification and functional analysis of non-coding regulatory small RNA FenSr3 in Bacillus amyloliquefaciens LPB-18
Source: PeerJ. 2023 May 15;11:e15236. doi: 10.7717/peerj.15236 (PMC10194069; doi:10.7717/peerj.15236)
Supplement: Supplemental Information 4 [file peerj-11-15236-s004.zip › KO/CK-vs-T1_map/map02026.html]

KEGG PATHWAY: Biofilm formation - Escherichia coli - Reference pathway


|  |  |
| --- | --- |
| **Biofilm formation - Escherichia coli - Reference pathway** |  |

[
Pathway menu
| Organism menu
| Pathway entry
| Show description
| User data mapping
]

Bacteria inhabiting a biofilm are protected from physical stress, antimicrobials, and the host immune system, and thereby cause severe medical, environmental, and technical problems. Biofilm formation in Escherichia coli is regulated by two inversely controlled transcriptional feedforward cascades, the FlhDC/FliA cascade and the sigmaS/MlrA/CsgD cascade, which are active in post-exponentially growing and in stationary phase cells, respectively. Their final outputs, flagella production or the synthesis of the biofilm matrix components curli and cellulose, are mutually exclusive due to complementary sigma factor requirements, the abundant DNA-binding regulator FliZ, and opposite regulation by c-di-GMP. The RNA-binding protein CsrA, a central component of the global regulatory system, inhibits synthesis of the poly-N-acetyl-glucosamine (PGA) exopolysaccharide and glycogen and stimulates the expression of the flagellar master regulator FlhDC. CsrA activity is counteracted by two sRNAs, CsrB and CsrC. The lower figure shows that FlhDC, sigmaS, and CsgD, the transcription factors that control large regulons involved in flagellar and biofilm formation, serve as regulatory hubs for multiple environmental signal integration with a series of sRNAs providing distinct inputs.

|  |  |  |
| --- | --- | --- |
| Reference pathway | 184% 150% 122% 100% 82% 67% 55% | 图片下载 |
